# Enhancing mathematical modeling competencies through AI-powered VR

**DOI:** 10.1371/journal.pone.0326440

**Published:** 2025-12-23

**Authors:** Nejla Gürefe, Hava Öksüz, Gülfem Sarpkaya Aktaş

**Affiliations:** 1 Faculty of Education, Mersin University, Mersin, Türkiye,; 2 Faculty of Education, Çukurova University, Adana, Türkiye; PLOS: Public Library of Science, UNITED KINGDOM OF GREAT BRITAIN AND NORTHERN IRELAND

## Abstract

**Background:**

Improving students’ problem-solving skills is one of the primary objectives of mathematics education. Problem-solving skills are closely related to the mathematical modeling process and the competencies required in this process, which are essential in various aspects of daily life.

**Method:**

The study was designed as a mixed design. A quasi-experimental design to examine the impact of an instructional model based on an artificial intelligence-supported virtual reality (VR) application on students’ modeling competencies for quantitative data and opinion and observation forms were used for qualitative data. These competencies included inductive, deductive, pragmatic, planned, and problem-solving thinking. The study involved 30 students from two 6th-grade classes at a Science and Art Center located in the western part of Turkey. One class served as the experimental group (f = 15) and engaged with the AI-pVR approach, while the other class served as the control group (f = 15) and followed the traditional teaching model. In the study, independent samples t-tests and ANCOVA were performed for quantitative analysis, and to check ANCOVA assumptions, normality, homogeneity of variances, linearity, and homogeneity of regression slopes were examined. Descriptive analysis was also performed for qualitative analysis.

**Results:**

The findings of this study revealed that the intervention had a significant large effect (η² = 0.37) on students’ mathematical modeling competencies. Among the components of modeling competence, understanding and simplifying the problem, mathematizing, working mathematically, interpreting, and verifying all exhibited significant large effects (η² = 0.33, 0.19, 0.32, 0.39, respectively), while defining the problem showed a moderate effect (η² = 0.15). The variability observed in some measurements may be attributed to limitations in the number of practice trials and the small sample size. However, the educational process carried out within the scope of this study has shown that students have made significant progress in their mathematical modeling skills. Students have stated that they have meaningfully grasped the basic steps of the modeling process, such as analyzing real-life problems, relating these problems to mathematical structures, creating models, solving the model they have created step by step, interpreting and verifying the results. In addition, students have developed a significant awareness in sharing their models with their friends and teachers, receiving meaningful feedback, developing new models for different life situations, and establishing connections between real life and mathematics.

**Conclusion:**

The results indicated that the integration of the AI-pVR instructional approach significantly improved students’ modeling competencies and related sub-dimensions. These sub-dimensions were problem understanding and simplification, mathematizing, working mathematically, interpreting, and verifying. Based on these findings, it is recommended that artificial intelligence applications, which can positively influence various areas such as competencies, should be incorporated into teachers’ lessons and even included in curriculum programs.

## 1. Introduction

In the 21st century, advancements in technology have significantly transformed the educational landscape, prompting innovations in mathematics curricula [1].Accordingly, mathematics education aims to link mathematics with daily life [2] and to enable students to mathematically interpret and analyse the events they encounter in daily life [3]. Given these reasons, mathematical modelling (MM) serves as a bridge that helps students connect mathematical concepts to real-world contexts [4]. Thus, it enables them to formulate and interpret everyday problems using mathematical reasoning [5,6] so that real world problems can be modelled and resolved [7–12].

Artificial intelligence-powered (AI-p) applications enhance instructional methodologies and the knowledge acquiring process [13–15]. While AI is helping educators in developing instructional resources, personalized digital ecosystems, and immersive educational spaces [14], VR provides an active learning environment and enables digital representation and interaction with situations encountered in daily life [16]. However, studies on how digital technologies can be used in MM education are limited [17,18]. Çevikbaş et al. [17] pointed out that the use of technology in the MM process is not sufficient. This situation reveals both the current lack of artificial intelligence applications in teaching environments and the need for the inclusion of these advanced tools in MM pedagogy. This study aims to address this gap by investigating optimal implementation strategies for artificial intelligence and virtual reality technologies in mathematical modeling instruction.

In the study, we aimed to reveal the effects of MM activities based on AI-powered VR (Alp-VR)applications on gifted students’ MM competencies. Accordingly, answers to the following questions will be sought:

1. Does AI-pVR have a statistically significant effect on students’ mathematical modeling competencies?
2. What are students’ opinions on mathematical modeling competence?

### 1.1. Theoretical foundations of mathematical modeling

**Fig 1:**
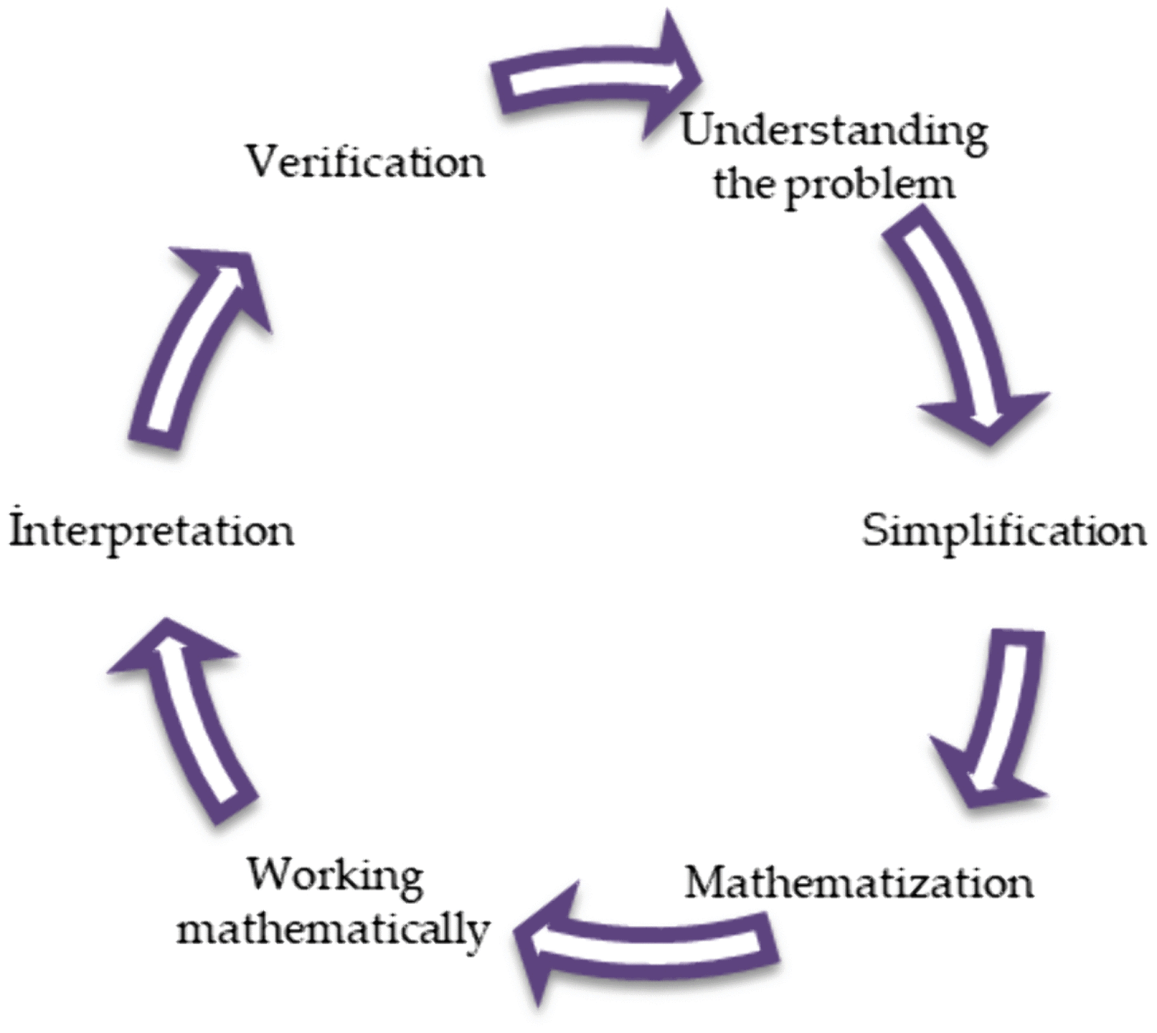
Mathematical modelling competencies [25].

Mathematical modeling represents a cyclical methodology for transforming authentic contextual problems into mathematical formulations, analyzing them through mathematical operations, and re-explaining solutions back into their original contexts [7,19]. This strategy includes constructing systematic representations to analyze and solve authentic contextual challenges [20]. Mathematical modelling emphasizes contextual problem-solving by anchoring tasks in authentic scenarios, which underscores its pedagogical value in education [21]. Kaiser [10] as well as Blum and Leiß [22] conceive mathematical modeling as a repetitive, circular process. Blum and Borromeo Ferri [23] further elaborate that this process initiates when learners engage with authentic, real-world problem situations. The process involves developing a mathematical representation through systematic simplification of the problem context. At the simplification stage, the student distinguishes the necessary and unnecessary parts of the problem situation [24]. The authentic scenario is subsequently formalized through mathematization, yielding an abstract mathematical representation. This constructed model undergoes systematic mathematical analysis to find the solutions, which are then interpreted and linked back to the original real-world context [23]. Consistent with this framework, mathematical modelling competencies can be conceptualized as a cyclical progression encompassing: (1) understanding the problem, (2) simplification, (3) mathematization, (4) working mathematically, (5) interpretation, and (6) verification as key phases of the modelling sequence (Fig 1). Contemporary research suggests these competencies may be evaluated either holistically across the entire cycle or through discrete component stages [25,26]. Besides the cognitive abilities, learner engagement and willingness to participate in modelling activities constitute equally vital dimensions.

Mathematical modelling competencies are the ability to progress in the model building process in a willing and purposeful way [27] and to carry out this process autonomously and consciously [28]. In fact, Maaß [27] stated that to fully understand mathematical modelling competencies, they should be directly related to the mathematical modelling process. Mathematical modelling competencies are of great importance in educational programmes and teaching in terms of supporting intelligence and social development as well as improving the interpretation process by solving real-life problems [29]. In this context, since gifted students have creative problem solving abilities and high motivation [30], they can be exposed to learning environments that will improve their MM competences.

Research has also focused on pedagogical design principles for mathematical modeling activities. Geiger et al. [31], building on Galbraith [32], proposed seven design principles for mathematical modeling activities [33]. In the present study, MM activities were developed by taking into account the design principles of MM activities developed by Geiger et al. [31] and Galbraith [32]. Lo, Huang & Cheung [33] adapted and articulated the design principles originally developed by Geiger et al. [31] and Galbraith [32] into the following framework: Nature of Problem (Principle 1: Problems should be open-ended and include both mathematical and non-mathematical knowledge), Relevance and Motivation (Principle 2: Problems should be connected to students’ real-life experiences and be part of their experiences), Accessibility (Principle 3: Problems can be approached mathematically, and the overall problem should suggest appropriate sub-questions), Feasibility of Approach (Principle 4: During the solution process, students should make the necessary assumptions using mathematics and gather the necessary data), Feasibility of Outcome (Principle 5: Students should perform the necessary mathematical solution and interpret the results), Feasibility of Evaluation (Principle 6: Students should be able to evaluate the mathematical accuracy and contextual relevance of the solution), and Didactical flexibility (Principle 7: Problems should be phrased sequentially, maintaining the integrity of real-life situations). As a result, MM activities designed based on these principles enable gifted students to reflect their unique problem-solving approaches and motivations in the modeling process. They can also provide students with higher-level mathematical thinking skills.

### 1.2. A conceptual framework for integrating digital technologies into mathematical modelling processes

**Fig 2:**
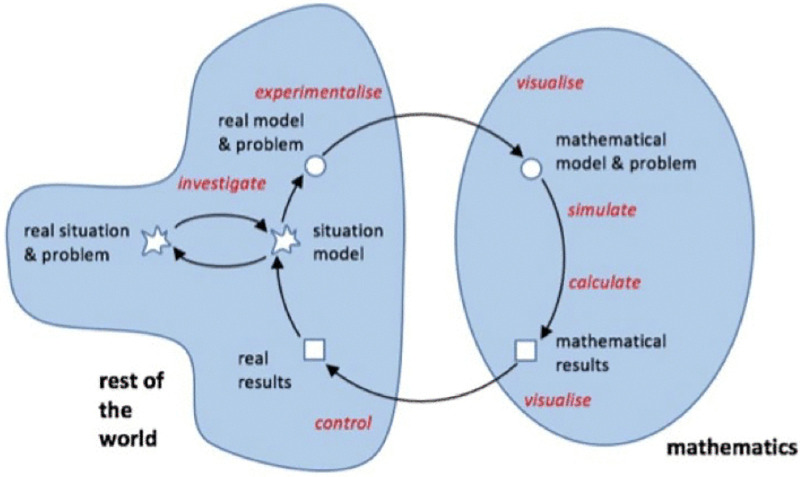
Usage of digital technology in mathematical modeling Blum & Leiß [22].

Digital technologies play a crucial role in mathematical modelling. Hıdıroğlu [34] emphasized that the integration of mathematical modelling and technology is essential for mathematics education to achieve its goals. In this sense, Blum and Leiß [22] provide a framework for how technology can be utilized in the mathematical modelling cycle (Fig 2).

Studies examining the integration of technology and mathematical modelling showed that technology was mostly used in the stages of creating a mathematical model, solving the mathematical model and revising the created mathematical model [35]. Given this context, it is imperative to consider both the potential advantages and constraints of using digital technologies in mathematical modelling education, as well as to explore their effectiveness in fostering students’ modelling skills.

Previous studies have shown that technology-supported learning environments can enrich modelling contexts [36], support both learners and educators in generating and investigating potential solutions to modelling tasks, offer insights and hands-on experiences with various types of problems [17], and facilitate the validation of models through real-world examples [37]. Moreover, digital technologies can be effectively integrated into various phases of the modelling cycle—such as simplification, mathematization, establishing connections, evaluation, interpretation, and validation [38]. Furthermore, Mousoulides et al. [39] stated that students’ use of technology in the learning environment helps them to recognize the modelling problem, actively engage with it, notice it, and support processes performed*.*

### 1.3. Use of VR in mathematical modelling

VR is an environment where the real world is transferred to the digital environment, consisting of three-dimensional models and designed based on individual interaction [40]. VR technologies enrich students’ learning environments, enabling them to gain new experiences in a virtual environment, conduct interactive studies [41], concretise abstract concepts [42], and improve their mathematical thinking skills and spatial abilities [43].

However, research on the use of VR in MM is limited [44]. Kim Pham et al. [44] examined the impact of VR on high school geometry teaching and reported that mathematical modeling is a fundamental skill in geometry. Their findings revealed that VR facilitates the understanding and visualization of geometric concepts and supports the process of creating mathematical models. They also found that students’ mathematical modeling skills improved while using VR. Based on these findings, they recommended using VR to develop students’ mathematical modeling skills in high school geometry teaching.

### 1.4. Use of AI in mathematical modelling

Artificial intelligence solutions grow ever more vital in education as a result of the development of digitalization. The introduction of AI into education help to create learning and teaching environments that satisfy educational demands [45] and fosters empirical research showing the effects of AI technologies. A primary objective of incorporating artificial intelligence into education is to investigate innovative instructional approaches and to foster the development of 21st-century cognitive and learning skills [46]. The integration of artificial intelligence technologies into mathematics education enhances students’ abstract thinking and problem-solving skills by offering dynamic visualizations of figures and mathematical concepts [47]. Since MM requires strong mathematical thinking and problem-solving skills, researchers have examined the MM capabilities of artificial intelligence tools such as GPTs. Spretitzer et al. [48] investigated the performance of GPT-3.5, GPT-4.0, and the more instructional GPT-MM model across several mathematical modelling situations. Their findings indicated that all versions exhibited fundamental competencies in mathematical problem solving within these contexts. However, their solutions vary according to the complexity of the MM tasks. Although GPT-4.0 and GPT-MM models showed rather great improvements in delivering sophisticated solutions with direction, they exprerienced problems in complex mathematical modelling situations. Furthermore, their results indicated that the clarity of the modelling tasks had a limited effect on performance, with mathematical and contextual complexity emerging as more significant determinants.

## 2. Materials and methods

### 2.1. Research design

This study employed a mixed method approach combining quantitative and qualitative data. This approach enhances the reliability and richness of the research [49]. An explanatory sequential mixed-methods design was used, in which quantitative data were collected and analyzed first, followed by qualitative data to provide deeper insights. In the quantitative phase of the study, a pretest-posttest quasi-experimental design was applied. Two sixth grade classes from a Science and Art Centre affiliated with a state institution were selected and randomly assigned to experimental and control groups. Since students were admitted to the Science and Art Center through an exam, they had comparable academic abilities. The study was conducted in the fall semester of the 2024 academic year. The intervention process was implemented in both groups by a mathematics teacher with documented expertise in artificial intelligence-supported pedagogical practices and virtual reality (VR)-based instruction. Before the intervention, students completed the Mathematical Modelling Competencies (MMC) Scale as a pretest; after the intervention, the same scale was readministered as a posttest to evaluate their progress.

**Fig 3:**
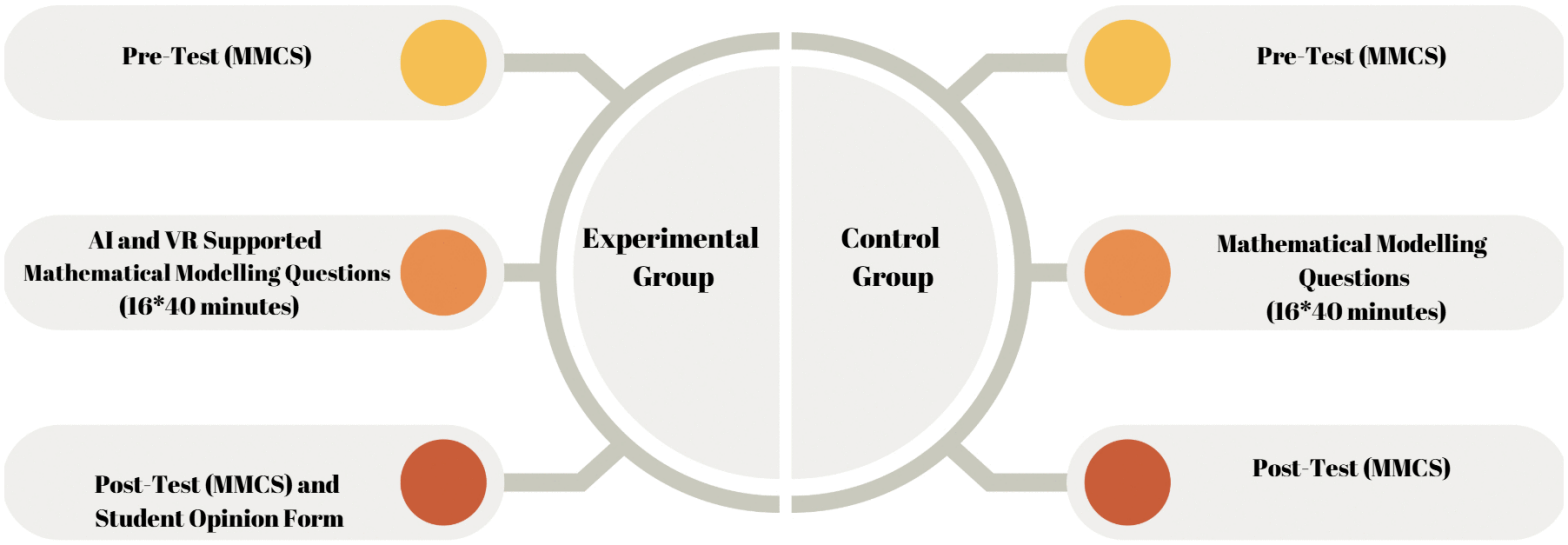
Research design of this study. MMCS: Mathematical Modeling Competencies Scale.

In the qualitative phase, a descriptive survey model was used to examine students’ views on their mathematical modelling competencies. A total of 13 students participated in the interviews. Although the experimental group consisted of 15 studentes, two rejected to participante.. Each interview form was distributed to the students and they were asked to complete it within one class period. After the intervention was completed, an interview form consisting of open-ended questions was distributed to collect data on ‘identifying real life problems, understanding and simplifying the problem, mathematising, working mathematically, interpreting and verifying’. These qualitative data aimed to provide a more in-depth perspective on students’ competence development. The research design is presented in Fig 3.

### 2.2. Study group

This research involved thirty 6th-grade students (aged 11–13 years, mean age = 12.03) enrolled at a Science and Art Center in western Turkey’s provincial capital. Participants were randomly assigned to the experimental group (n = 15; 11 boys, 4 girls) and the control group (n = 15; 8 boys, 7 girls). These students receive out-of-school enrichment education from Science and Art Centres which are institutions for gifted students in Türkiye that admit students through an examination. All participants were enrolled in ongoing computer literacy courses at the Science and Art Center, ensuring baseline technological competency. Prior to the study, written informed consent was obtained from students and their parents/guardians, with comprehensive explanations provided about the use of artificial intelligence and virtual reality technologies in the study. In addition, the school administration granted approval for voluntary participation in the study. Participants were included in the study between 15 October and 30 November 2024.

### 2.3. Data collection tools

Quantitative phase: The MMC scale was applied as a pre-test and post-test to evaluate students’ mathematical modelling competencies before and after the intervention. The Mathematical Modelling Competence (MMC) Scale was adapted from Özbek and Köse’s [50] study. The scale consists of 31 items grouped into five factors. The first factor includes 3 items assessing students‘ability to identify and relate real-life problems; the second factor includes 7 items measuring students’ ability to understand and simplify problems; the third factor includes 3 items assessing mathematisation processes; the fourth factor includes 6 items focusing on mathematical work; and the fifth factor includes 12 items measuring interpretation and verification skills. All items were rated using a five-point Likert scale ranging from 1 (Strongly Disagree) to 5 (Strongly Agree). The internal consistency of the scale was assessed using the KR-20 coefficient and high reliability coefficients were obtained:.811 for the first factor,.900 for the second factor,.883 for the third factor,.820 for the fourth factor and.927 for the fifth factor.

Qualitative phase: An opinion form was prepared to obtain students’ views on the subcategories of mathematical modeling competence, “Identifying real-life problems, understanding and simplifying the problem, mathematizing, working mathematically, interpreting, and verifying.” It was reviewed by two faculty members with expertise in mathematics education who assessed the interview questions for understandability, language, and suitability for the sub-dimensions. Following their feedback, some statements were revised or simplified. For example, in the first draft, the question, “Did your self-confidence in solving mathematical problems increase after this exercise?” was supplemented with the questions, “a) If yes, please explain why?” and “b) If no, please explain why.” As a result of the expert opinion, the form consisting of thirteen questions was finalized and developed. This form was completed by the students within one class period at the end of the modeling process.

Data from the pre-test, post-test, and interview forms were collected in the fall semester of 2024 and subsequently analyzed. All data were anonymized to protect participant confidentiality.

### 2.4. Ethical considerations

This study was conducted in accordance with the ethical principles governing research in educational settings. The study was conducted in accordance with the Declaration of Helsinki, and approved by Ethics Committee of Mersin University (protocol code E-52793531-050.04-2884893 and date of Approval 23/09/2024).

In data collection process, written informed consent was obtained from the parents or legal guardians of all student participants. The participants and their families were thoroughly informed about the voluntary nature of their involvement and assured that they could withdraw from the study at any time without facing any adverse consequences. Participation was entirely voluntary, and all ethical guidelines related to confidentiality and autonomy were strictly adhered to.

### 2.5. Data collection process

In both groups, instruction was delivered by a teacher who was a part of the research team. This educator held a master’s degree and had completed various training courses (including “the use of metaverse and artificial intelligence tools”, “digital transformation in measurement and evaluation: artificial intelligence”) in the application of artificial intelligence (AI)-supported tools and virtual reality (VR) technologies, thereby demonstrating their expertise. Virtual Reality (VR) applications, in this study, refer to 360-degree rotatable video environments designed to offer users an immersive and interactive virtual experience [51]. The intervention lasted 18 lesson. The pre-test was administered in the first lesson, and the post-test was administered in the last lesson (18th lesson). The teaching process was carried out during the 16 lesson sessions between the pretest and posttes. The instructional process for both groups commenced simultaneously. Each session lasted approximately 40 minutes, ensuring consistency in instructional time across the groups. In preparing the mathematical modeling questions for both groups, the ethnomodeling approach was used to enable students to engage with the problems appropriate to their own cultural structures. Ethnomodeling method is defined as the study of mathematical phenomena that incorporate cultural expressions intoto modeling questions [5,45]. Ethnomodeling questions are a powerful tool for connecting to students’ real life experiences [5]. In addition, the modeling questions included places near the students’ region with historical and natural features that could be explored using VR goggles. The modeling questions were designed based on the mathematical modeling design principles developed by Geiger et al. [31] and Galbraith [32] (Nature of problem, Relevance and motivation, Accessibility, Feasibility of approach, Feasibility of outcome, Feasibility of evaluation, and Didactical flexibility). In this context, mathematical modeling questions supported students’ daily life experiences, cultural contexts, and mathematical knowledge and skills. Furthermore, the adaptability of the questions to different situations provided strong examples of didactic flexibility and evaluative principles. Therefore, the modeling questions encouraged the development of students’ mathematical modeling competencies. An evaluation of the mathematical modeling questions based on these design principles is presented in Table 1.

**Table 1:** Analysis of mathematical modeling questions within the framework of mathematical modeling design principles by Geiger et al. [31]; Galbraith [32].

Principle	Modelling Problem	Description
Principle 1. Nature of problem	Clandras Bridge, Glass Terrace (Ulubey Canyon), Bin Tepe Tumuli, Ephesus Antique City	The problem is open-ended in terms of approaches to mathematical modeling.
	Tasyaran Valley, Güney Waterfall, Kula Fairy Chimneys, Pamukkale Traventers	The problem is open-ended in terms of making assumptions.
Principle 2. Relevance and motivation	Clandras Bridge, Glass Terrace (Ulubey Canyon), Tasyaran Valley	They are local and cultural heritage, found in students’ daily lives and their surroundings.
	Pamukkale Traventers, Güney Waterfall,	They are naturally occurring places, familiar to students and known as tourist attractions.
	Bin Tepe Tumuli, Ephesus Antique City	They are historical and cultural heritage, familiar to students.
Principle 3. Accessibility	Clandras Bridge	The amount of glass required as the bridge length changes
	Pamukkale Traventers	Change in the area covered by the tumuli due to changes in width
	Kula Fairy Chimneys	Calculating how height changes over time
	Güney Waterfall	Annual change in height depending on the year
	Glass Terrace (Ulubey Canyon)	The number of uses of rectangular glass according to changes in area
	Tasyaran Valley	Erosion rate at different lengths and depths
	Bin Tepe Tumuli	What percentage of the original tumuli remains?
	Ephesus Antique City	Calculating the shortest distance based on the number of breaks and walking speed
Principle 4. Feasibility of approach	Clandras Bridge, Pamukkale Traventers, Glass Terrace (Ulubey Canyon	Area problem
	Kula Fairy Chimneys, Güney Waterfall, Tasyaran Valley, Bin Tepe Tumuli)	Ratio-proportion problem
	Ephesus Antique City	Time problem
Principle 5. Feasibility of outcome	Clandras Bridge	Adaptability of the model to bridges of different lengths
	Pamukkale Traventers,	The result must be understandable as the number of football fields and a concrete comparison must be made.
	Kula Fairy Chimneys	Interpreting the results by relating them to the erosion process
	Güney Waterfall	Interpreting the elevation over time by considering annual increases
	Glass Terrace (Ulubey Canyon)	Calculating the number of tumuli in a contextual manner
	Tasyaran Valley	The results can be interpreted in terms of natural processes.
	Bin Tepe Tumuli	Understanding the change in the number of existing tumuli over time and the need for preservation.
	Ephesus Antique City	Determining the shortest route by calculating the travel time for each route.
Principle 6. Feasibility of evaluation	Clandras Bridge, Pamukkale Traventers, Glass Terrace (Ulubey Canyon)	It can be mathematically controlled and recalculated at different scales.
	Kula Fairy Chimneys, Güney Waterfall, Bin Tepe Tumuli, Tasyaran Valley	It can be controlled by changing the duration.
	Ephesus Antique City	It can be checked with different speed/break values.
Principle 7. Didactical flexibility	Clandras Bridge	First, ask about the area, then the amount of glass, and its adaptability to bridges of different lengths.
	Pamukkale Traventers	First, choose the average width, then calculate the area and compare it to a football field.
	Kula Fairy Chimneys	Asking how to calculate erosion in different years using existing data first, then using this data.
	Güney Waterfall	Asking how the annual increase changes over time and how to adapt it to other waterfalls
	Glass Terrace (Ulubey Canyon)	First, relate the terrace area to the glass dimensions, then ask about adapting it to different sizes.
	Tasyaran Valley	First, ask about valley erosion over time, then ask about adapting it to other valleys.
	Bin Tepe Tumuli	Asking how to convert the current number to a percentage, then calculate the annual decrease and make a prediction for 10 years from now.
	Ephesus Antique City	Asking how to calculate the route first, then calculate the route by adding stopover times, and then make a comparison.

Table 1 shows that the mathematical modeling questions were suitable in terms of students’ relevance to local cultural and natural heritage (principles 1 and 2); sub-questions could be asked that were appropriately to the overall problem (principle 3); calculations could be made using previously learned concepts such as ratio-proportion calculations, and time calculations (principle 4); the results obtained were understandable and interpretable within the context (principle 5); calculations can be made to explore variability (principle 6); and the results could be adapted to different situations and evaluated (principle 7). Thıs, the mathematical modeling questions prepared were in line with the seven design principles proposed by Geiger et al. [31] and Galbraith [32]. Details about the modeling problems are given in Table 2.

**Table 2:** Modeling problems.

Problem	Problem Topic
Clandras Bridge	The railings on both sides of the Clandras bridge are to be replaced with glass railings with a height of 1 meter. Students are tasked with determining the amount of glass railing, in square meters, required for the design*. (Area problem) (See* Fig 4 *for the visual representation).*
Pamukkale Traventers	Information about Pamukkale traventers was given. The students were tasked with estimating the total area of the Pamukkale travertines and determining how many football fields it would correspond to. *(Area problem) (See* Fig 5 *for the visual representation).*
Kula Fairy Chimneys	Information was given about fairy chimneys and how much erosion they suffered on average. The students were asked to determine the projected average height of the fairy chimneys in the valley after 20 years*. (Ratio-proportion problem) (See* Fig 6 *for the visual representation).*
Güney Waterfall	Data about the waterfall are given. Students are asked to make predictions about what the height of the waterfall will be after 4 years. *(Ratio-proportion problem) (See* Fig 7 *for the visual representation).*
Glass Terrace (Ulubey Canyon)	The total square meters of the glass terrace is given. Using glass rectangles given in certain sizes, students are asked approximately how many glass rectangles they should use to cover this glass terrace and how they should place them. *(Area problem) (See* Fig 8 *for the visual representation).*
Tasyaran Valley	Information about the formation of Taşyaran valley is given. Students are asked to make a prediction about the depth of the Taşyaran valley 1000 years later and to elaborate on how they made this prediction. *(Ratio-proportion problem) (See* Fig 9 *for the visual representation).*
Bin Tepe Tumuli	Information was given about how the tumuli were destroyed over time. Students are asked to predict how many tumuli will remain in 10 years and to suggest what should be done to protect them. *(Ratio-proportion problem) (See* Fig 10 *for the visual representation).*
Ephesus Antique City	Different routes to visit the ancient city of Ephesus, the length of the routes and the duration of the stops are given. An average walking speed is also given. Students are asked to predict which route will take the shortest time with breaks*. (Time problem) (See* Fig 11 *for the visual representation).*

**Fig 4:**
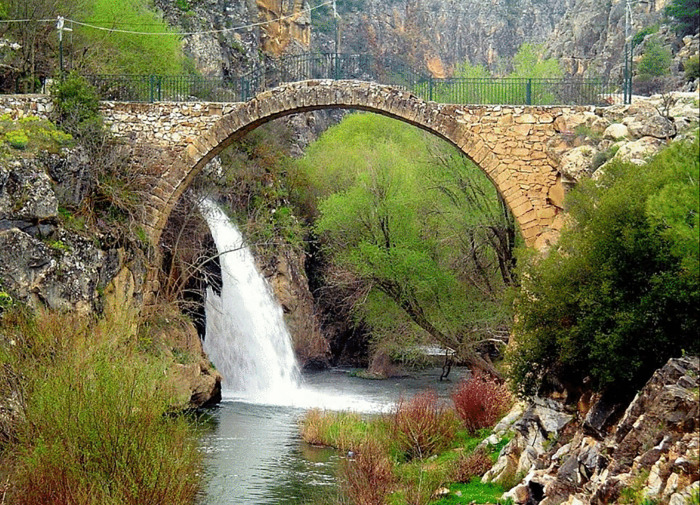
Clandras Bridge image.

**Fig 5:**
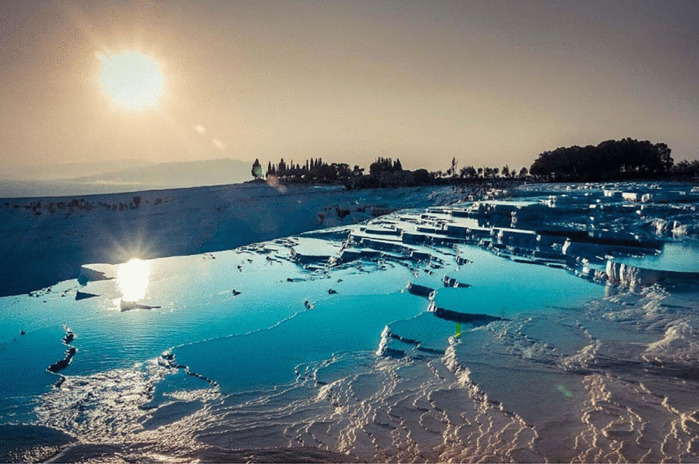
Pamukkale Traventers image.

**Fig 6:**
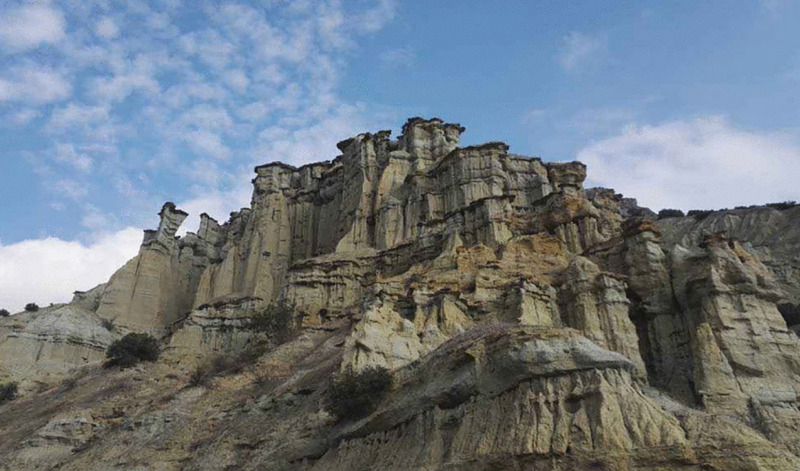
Kula Fairy Chimneys image.

**Fig 7:**
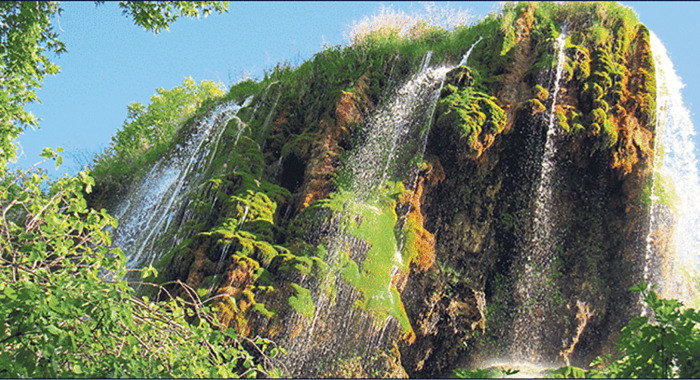
Guney Waterfall image.

**Fig 8:**
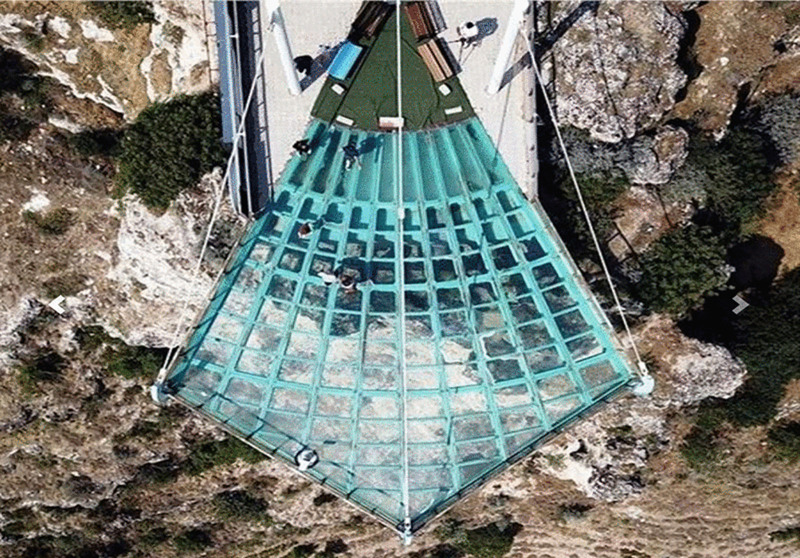
Glass Terrace (Ulubey Canyon) image.

**Fig 9:**
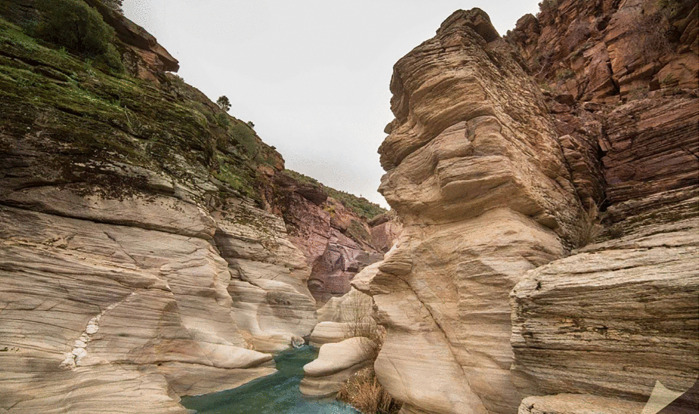
Tasyaran Valley image.

**Fig 10:**
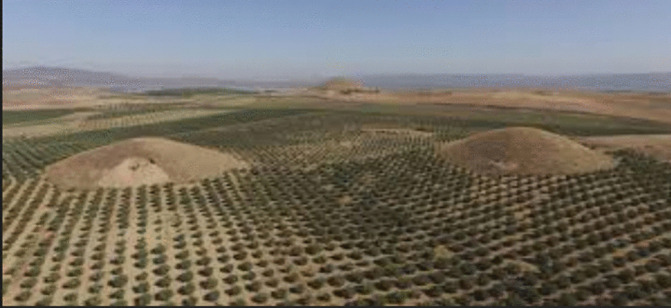
Bin Tepe Tumuli image.

**Fig 11:**
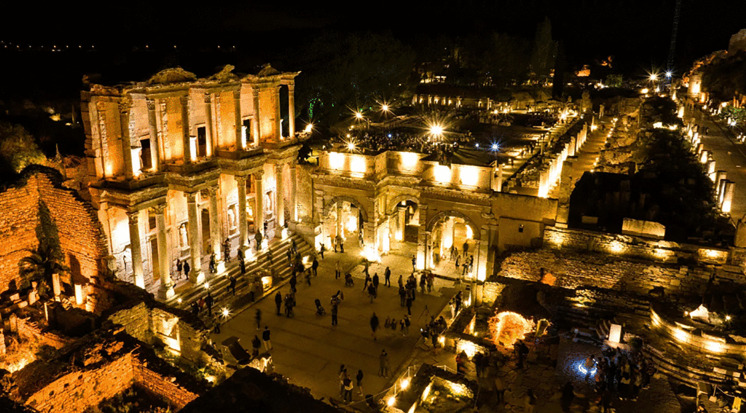
Ephesus Antique City image.

Before the administration, the questions were reviewed by a mathematics teacher and two subject-matter experts involved in the study. Based on their feedback, the questions were revised and then implemented. The students in both groups solved the questions in teams of three. Research has shown that collaborative work positively impacts the process of solving modeling questions [52]. The modeling questions were designed in alignment with the theoretical framework, considering the modeling cycle [27] and modelling competencies [53] based on the stages of the modelling process.

#### 2.5.1. Week 1: Pre-testing and Information.

The MMC scale was administered to students in both the control and experimental groups as pretests to evaluate their baseline mathematical modelling competencies and thinking skills. The students in both groups were divided into teams of three. The experimental group was informed that each team would be provided with a tablet or laptop computer. In addition, small-scale sessions were conducted to familiarize the students with the use of VR goggles (Meta Quest 3-128 Gb, Meta Quest 2, VR-Box 3D). In contrast, the control group was informed that they would complete the tasks without any technological devices.

#### 2.5.2. Week 2–7: VR and AI-supported mathematical modeling vs. traditional problem-solving.

In the experimental group, students were provided with worksheets containing mathematical modeling problems related to real-life scenarios, such as the Clandras Bridge, Pamukkale Travertines, Kula Fairy Chimneys, Güney Waterfall, Glass Terrace (Ulubey Canyon), Taşyaran Valley, and Bin Tepe Tumuli. These problems were designed to integrate mathematical concepts with cultural and geographical contexts, fostering a deeper connection between mathematics and real-world applications.

**Fig 12:**
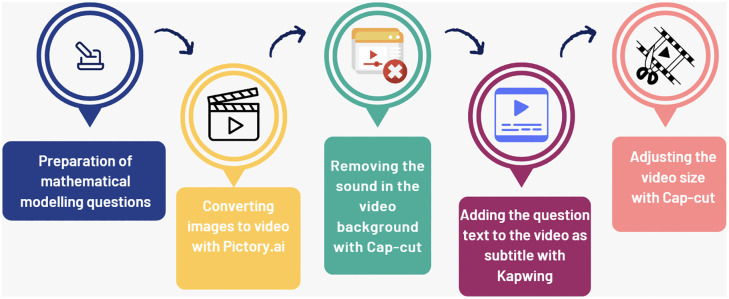
Video conversion of modelling questions.

After receiving the worksheets, students were shown videos that explained the mathematical modeling problems and provided background information about the locations mentioned in the problems. The AI tools used to create the videos were used solely by the researcher, and the students did not interact directly with them. Students simply watched the videos. The teacher’s purpose in using AI tools here was to help students better understand the context of mathematical modeling questions and the context between them, allowing them to make connections between real life and mathematics. Thus, these videos were intended to enable students to more easily adapt to the questions. Therefore, AI tools were part of the process and were used for material purposes. After preparing the modeling questions, the researcher teacher first obtained images of the places mentioned in the questions from Google Images, https://www.pexels.com/, https://unsplash.com/image/stock, www.freepik.com, and www.pixabay.com. The images of the places mentioned in each question were then compiled using the Pictory.ai(free) artificial intelligence tool to create a video. As audio was automatically added to videos created by the Pictory.ai (free) artificial intelligence tool, we used the Cap-cut (free) tool to remove this background audio. This tool completely removed the background audio. The text of the modeling questions was then added as subtitles to the videos using the Kapwing application. Finally, to eliminate inconsistencies between subtitles and visuals in some videos, timing adjustments were made using the Cup-cat (free) application, producing the final video presentations of the modeling questions. A visual representation of our work is shown in Fig 12. A sample modelling question video is available at: https://drive.google.com/drive/folders/1OXUsiNxFGuxkvFzEL0O71ruUUYu924vm?usp=sharing.

**Fig 13:**
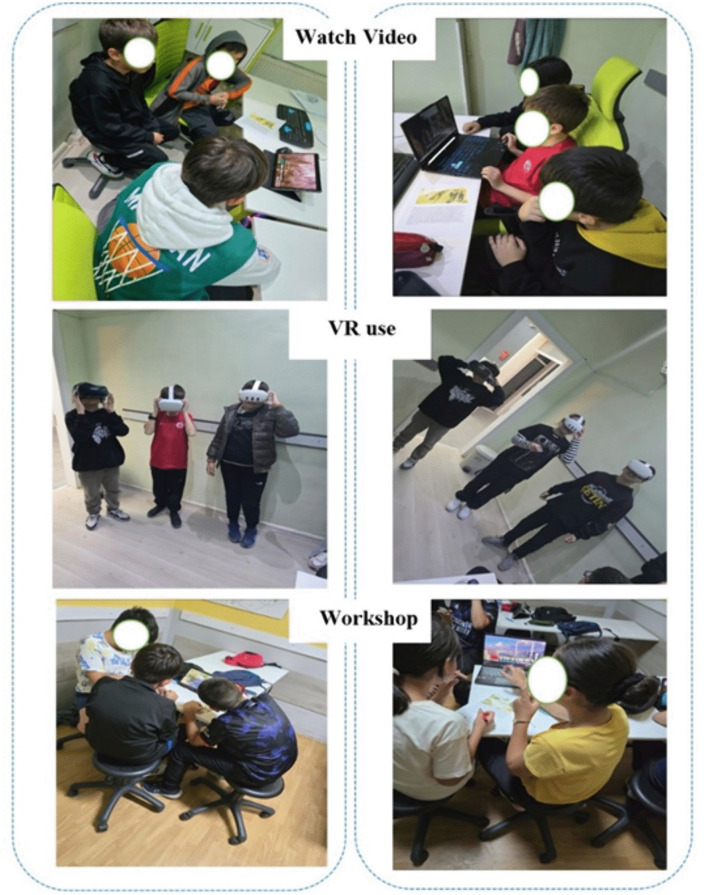
Classroom activities.

Following the video presentations, students were asked to solve the mathematical modeling problems. To enhance their understanding and engagement, they were provided with VR goggles to observe and explore the locations mentioned in the problems. The VR experience allowed students to visually immerse themselves in the scenarios, enabling them to better comprehend the context of the problems and apply mathematical concepts more effectively (see Fig 13).

The students in the control group also collaborated in groups to solve the same problems. They discussed their solutions and received feedback from the teacher, but without the technological support provided to the experimental group.

#### 2.5.3. Week 8: Post-testing.

Both groups completed the MMC scale as post-tests to evaluate the impact of the intervention on their mathematical modelling competencies and thinking skill. In addition, the students in the experimental group were asked to complete the opinion form about MMC within one lesson period. Data were collected from 13 of the 15 experimental group students, as 2 students were absent during the qualitative data collection phase.

*Data Analysis:* In this study, ANCOVA was utilized to test the research questions and examine whether there were significant differences between the control and experimental groups regarding the impact of the instructional model with AI-VR on the total scores of MMC. To evaluate the equivalence of the pre-test scores between the experimental group (EG) and the control group (CG), an independent samples t-test was performed, which showed no significant difference between the groups (p > 0.05). Before conducting the ANOVAs, the assumption of normality was assessed by examining the skewness, kurtosis of the data distribution and Shapiro-Wilk test. Data exhibiting skewness and kurtosis values between +1.5 and −1.5 were deemed acceptable and considered to follow a normal distribution [54] and a p-value greater than 0.05 in the Shapiro-Wilk test indicated normality. Additionally, the assumption of homogeneity of variance was evaluated using Levene’s test. The p-value above 0.05 in the Levene test indicated homogeneity of variance. The assumption of linearity was verified by analyzing the correlation between the covariate and the dependent variable to confirm a significant relationship. The equality of regression slopes was evaluated through ANCOVA, with slopes deemed equal if p > 0.05. After confirming all assumptions, ANCOVA was performed to determine if there were significant differences in post-test scores among the groups. The effect size was calculated using eta squared (η²) to assess the magnitude of the observed differences. If the calculated eta squared value is between.01 and.06, the effect size is “small”; if it is between.06 and.14, it has a “medium” effect; if it is.14 and above, it has a “large” effect [55].

Descriptive analysis was used to analyze the qualitative data. Codes were identified and the combined into specific themes, and themes were combined into specific categories. To ensure validity in the data analysis, the opinions of two experts were sought during the coding process. The themes were revised based on their opinions. Participants were asked whether they confirmed their views. A clear contextual report was prepared, supported by direct quotes from the participants’ statements. To ensure reliability, the data obtained in the study was first analyzed by the first researcher and conducted descriptive analysis. The same data set was then independently analyzed by the second researcher and conducted content analysis. To ensure confirmability, the coding reliability between the two researchers was determined to be 85%. Any disagreements that arose during the coding process were resolved through discussion, and consensus was reached on all codes. At the end of this process, the required level of reliability in qualitative analysis was achieved.

## 3. Results

### 3.1. Quantitative findings on modeling self-efficacy

#### 3.1.1. Descriptive statistics results.

**Fig 14:**
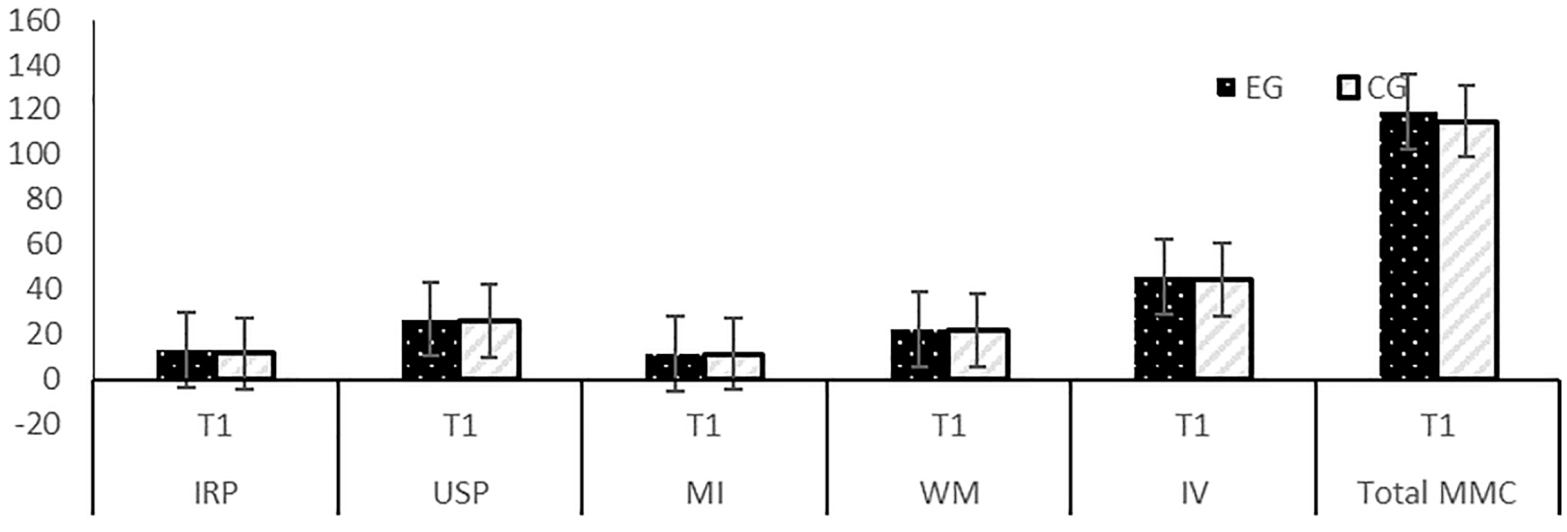
The pre-test (T1) results of EG and CG for MMC (sub-dimensions: IRP, USP, MI, WM, IV).

**Fig 15:**
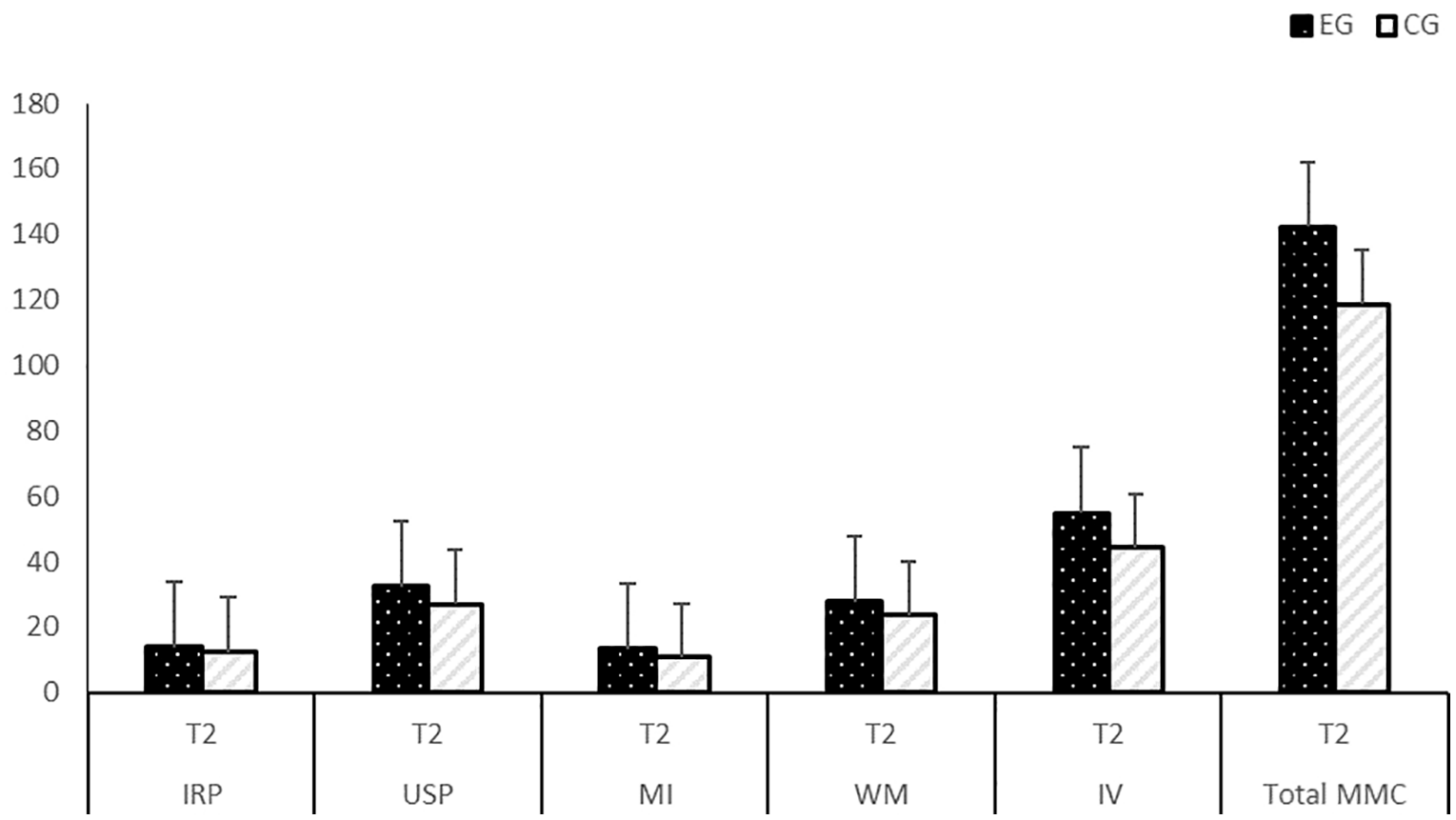
The post-test (T2) results of EG and CG for MMC (sub-dimensions: IRP, USP, MI, WM, IV).

The results for MMC and its sub-dimensions are presented in Tables 3 and 4 and Figs 14 and 15. No differences were identified between the EG and CG at the time of study enrolment (T1) in terms MMC outcomes and their variables (Table 3).

**Table 3:** Independent samples t-test scores for MMC and its sub-dimensions.

MMC	Groups	n	T1	T2		
			(Mean ± S.D)	(Mean ± S.D)	t	p
IRP	EG	15	12.73 ± 2.15	14.26 ± 1.03	−1.209	0.237
	CG	15	11.60 ± 2.92	12.13 ± 3.02		
USP	EG	15	26.86 ± 5.68	32.40 ± 2.22	−0.488	0.629
	CG	15	25.86 ± 5.54	27.00 ± 5.02		
MI	EG	15	11.53 ± 2.26	13.40 ± 1.29	−0.349	0.730
	CG	15	11.26 ± 1.90	11.26 ± 2.89		
WM	EG	15	22.46 ± 4.03	27.80 ± 2.21	−0.443	0.662
	CG	15	21.73 ± 4.99	23.46 ± 4.47		
IV	EG	15	45.60 ± 8.16	54.93 ± 3.97	−0.416	0.680
	CG	5	44.40 ± 7.61	44.20 ± 8.77		
Total MMC	EG	15	119.20 ± 20.71	142.80 ± 8.75	−0.591	0.559
	CG	15	114.86 ± 19.41	118.06 ± 21.31		

T1: pretest; T2: postest; MMC: Mathematical Modeling Competencies, IRP: Identifying the Real-life Problem, USP: Understanding and Simplifying the Problem, MI: Mathematizing, WM: Working Mathematically, IV: Interpretation and Validation.

**Table 4:** Adjusted and Unadjusted means and variability of engagement on the two groups.

			Unadjusted		Adjusted		
Variable	Groups	N	M	SD	M	SE	F(p)
IRP	EG	15	14.26	1.03	13.96	0.48	4.961**
	CG	15	12.13	3.02	12.43	0.48	
USP	EG	15	32.40	2.22	32.31	0.99	13.813**
	CG	15	27.00	5.02	27.08	0.99	
MI	EG	15	13.40	1.29	13.35	0.55	6.659**
	CG	15	11.26	2.89	11.31	0.55	
WM	EG	15	27.80	2.21	27.65	0.80	12.690**
	CG	15	23.46	4.47	23.61	0.80	
IV	EG	15	54.93	3.97	54.85	1.77	17.718**
	CG	15	44.20	8.77	44.27	1.77	
Total MMC	EG	15	142.80	8.75	142.29	4.11	16.497**
	CG	15	118.06	21.31	118.57	4.11	

The means of students’ MMC were similar across the groups (p > 0.05).

#### 3.1.2. Findings related to MMC and Its sub-dimensions post-test results of experimental and control groups.

The assumptions of homogeneity of variance, normal distribution of the data, linearity, and homogeneity of regression slopes were tested before performing ANCOVA. The Shapiro-Wilk test and skewness and kurtosis values were used to evaluate normality. For the MMC variable, the p-value in the Shapiro-Wilk test was more than 0.05, and each group’s skewness and kurtosis values ranged from −1.58 to 0.41 and −1.52 to 2.74, respectively, which showed that the assumption of normalcy was satisfied. Levene’s test for determining homogeneity of variance was not violated (p > 0.05). The linearity assumption was evaluated by examining scatterplots of the covariates against the dependent variables. The patterns observed indicated approximately linear relationships, which supported the validity of the linearity assumption.

To assess the homogeneity of regression slopes, the interaction between the covariate and the group was examined. The analysis indicated that the assumption of homogeneous regression slopes was upheld for the total MMC and all its subscales (p > 0.05). Specifically, the interaction between pre-test scores and group membership on post-test scores for the subscales (IRP, USP, MI, WM, IV) and the total MMC was not significant [F(1, 26) = 2.385, p > 0.05; F(1, 26) = 0.261, p > 0.05; F(1, 26) = 0.018, p > 0.05; F(1, 26) = 0.211, p > 0.05; F(1, 26) = 0.077, p > 0.05, F(1, 26) = 0.037, p > 0.05, respectively]. These results suggest that the regression slopes for predicting post-test scores based on pre-test scores were equivalent across both the experimental and control groups. ANCOVA was then used, since these findings suggested that the assumptions were met. The ANCOVA results comparing total MMC and its variables’ scores of students were summarized in Tables 4 and 5.

**Table 5:** Sizes of partial eta squared (η2).

Variable	Groups	Partial Eta Squared (η2)
IRP	EG	0.155
	CG	
USP	EG	0.338
	CG	
MI	EG	0.198
	CG	
WM	EG	0.320
	CG	
IV	EG	0.396
	CG	
Total MMC	EG	0.379
	CG	

ANCOVA was conducted to determine whether there was a significant difference in the development of MMC between the experimental and control groups. Table 4 and Fig 15 show that after controlling for pre-test scores as covariates, there was a significant difference between the total post-test IRP scores of the two groups. Specifically, students in the EG (M = 13.96; SE = 0.48) outperformed those in CG (M = 12.43; SE = 0.48) in the development of their IRP [F(1, 27) =4.96, p < 0.01, η2 = 0.15]. The AI-pVR approach accounted for 15% of the variance in students’ ability to identify real-life problems, which indicated that students in the experimental group outperformed those in the control group. This represented a large effect size, as eta squared values of 0.14 and above are considered large [55]. Besides, there was a significant difference between the total post-test USP scores of the two groups. Specifically, students in the EG (M = 32.31; SE = 0.99) outperformed those in CG (M = 27.08; SE = 0.99) in the development of their USP [F(1, 27) = 13.81, p < 0.01, η2 = 0.33]. The AI-pVR approach accounted for 33% of the variance in students’ understanding and simplifying problems, indicating that students in the experimental group outperformed those in the control group, representing a large effect size [55]. In addition, a significant difference was found between the total post-test MI scores of the two groups. Specifically, students in the EG (M = 13.35; SE = 0.55) outperformed those in CG (M = 11.31; SE = 0.55) in the development of their MI [F(1, 27) = 6.65, p < 0.01, η2 = 0.19]. The AI-pVR approach explained 19% of the variance in students’ mathematizing abilities, and students in the experimental group outperformed those in the control group (large effect size) [55]). Also a significant difference emerged between the total post-test WM scores of the two groups. Particularly, students in the EG (M = 27.65; SE = 0.80) outperformed those in CG (M = 23.61; SE = 0.80) in their WM development [F(1, 27) = 12.69, p < 0.01, η2 = 0.32]. The AI-pVR approach accounted for 32% of the variance in students’ ability to work mathematically, and students in the experimental group outperformed those in the control group (large effect size) [55]. Finally, there was a significant difference between the post-test IV scores of the two groups. Specifically, the results revealed that students in the EG (M = 54.85; SE = 1.77) outperformed those in CG (M = 44.27; SE = 1.77) in their IV development [F(1, 27) = 17.71, p < 0.01, η2 = 0.39]. The AI-pVR approach explained 39% of the variance in students’ interpretation and validation abilities, and students in the experimental group outperformed those in the control group (large effect size) [55].

### 3.2. Qualitative findings on modeling competencies

The qualitative findings regarding students’ modeling competencies are summarized in Table 6.

**Table 6:** Categories and sub-categories for qualitative data.

*Categories*	*Sub-Categories*	*f (%)*
Identifying the Real-life Problem	Environmental and physical problems	6 (%46)
	Observing their environment, looking carefully, questioning and choosing problems suitable for mathematical modeling	4 (%30)
	Transportation and time management problems	2 (%15)
	Economic and decision-making problems	2 (%15)
Understanding and Simplifying the Problem	Understanding the problem (carefully to read the problems, distinguished the given and requested information, underlined the important parts and took notes when necessary)	7 (%54)
	Simplifying the problem (to eliminate unnecessary information and focus only on the elements necessary for the solution)	4 (%30)
	Developing hypotheses (to complete the missing or uncertain information with observations and logical assumptions, using visualization tools such as tables, graphs, and figures)	2 (%15)
Mathematizing	Using table	12 (%92)
	Using figures and graphs	8 (%61)
	Using technology	2 (%15)
Working Mathematically	Model solution process and	9 (%69)
	Mathematical association strategies	3 (%23)
Interpretation and Validation	Comparing their models with data obtained from real life and explaining it to their friends or teachers	10 (%77)
	Reviewing the solution steps from beginning to end, checked the operations or tried to fix the errors by correcting the incorrectly used formulas	9 (%69)
	Paying attention to the daily equivalents of the numbers, operations, and expressions	8 (%61)
	Correcting the errors by redoing the entire solution	4 (%30)

The findings regarding the categories and subcategories in Table 6 are discussed below.

#### 3.2.1. Identifying the real-life problem.

Students’ ability to identify real-life problems was analysed under four main themes based on participant statements. First, six students identified environmental and physical problems theme by emphasizing issues related to cleanliness, waste, changes in natural resources and physical conditions in the school environment (e.g., classroom temperature, bag weight). After the training, S4 stated, “*After the training, I became more sensitive to environmental problems. For example, the decrease in the speed of the waterfall and the pollution in the environment showed the seriousness of this problem*.”. S13 stated, “*I noticed that there was a lot of paper waste at school. I will produce solutions such as reducing paper use and sharing course materials in a digital environment.”.* Under problem recognition approaches theme, the participants defined problems through strategic means such as observing their environment, looking carefully, questioning and choosing problems suitable for mathematical modeling. This theme showed that the participants’ relationship with real life is multidimensional and that they integrated mathematical thinking into various aspects of daily lif. For example, S2 said *“I observe my surroundings, and as a result of my observations, I identify things that draw my attention.”* and S3 stated*”I look at my surroundings carefully.”.* The third theme (two students) focused on economic and decision-making problems. The participants highlighted problems through price comparisons, consumer preferences and economic factors affecting social life. For instance, S7 explained, *“I noticed that the same product has different prices in the markets. I can determine which market is cheaper.”*. Finally, under the transportation and time management problems theme, the participants defined situations that directly affected their daily lives such as traffic congestion, inadequate bus schedules and loss of time as problems. For eample, S1 said *“I examine my daily life. I look at where I waste time, what takes up most of my time.”.* Participant statements indicated that their awareness of identifying real-life problems increased thanks to this training.

#### 3.2.2. Understanding and simplifying the problem.

Based on participant statements, the process of understanding and simplifying real-life problems was analysed under three main sub-themes. In the first theme, understanding the problem, seven participants indicated that they carefully read the problems, distinguished the given and requested information, underlined the important parts and took notes when necessary. This shows that they developed a conscious approach to understanding the problem correctly and identifying the data necessary for the solution through the intervention. For example, S12 said that: “*First, I try to understand what the question is telling me. Then I determine the missing or unclear parts in the question*.”. Similarly, S3 expressed, *“I first read the problem slowly and underline the important parts. Once I understand the question, I cross out the unnecessary parts.”* and S5 stated, “*I read the question carefully and write down what is given*.”. In the second theme, simplifying the problem, it was found that the participants tended to eliminate unnecessary information in order to simplify the problems and focus only on the elements necessary for the solution. In this simplification process, breaking the problem into smaller parts and proceeding step by step were among the most frequently preferred methods. For example, S7 stated: “*I read thoroughly, determine what is given and what is requested, and remove the useless ones from the given ones.”.* In addition, S2 said: “*I remove unnecessary details and leave out important things. This way, the solution becomes clearer.”.* Finally, in the developing hypotheses sub-theme, participants stated that they completed the missing or uncertain information through observations and logical assumptions, and also made the problem more understandable by using visualization tools such as tables, graphs, and figures. These findings reveal that the intervention was effective in structuring the participants’ problem-solving skills and developing a systematic thinking process, and that the participants made simplifications based on their observations and logical assumptions in situations where all the information was not available in real life. For example, S4 explained, “*When I create my assumptions, I first simplify the problem. It can be difficult to think of every detail*.”

#### 3.2.3. Mathematizing.

The participants’ statements regarding the tools they used in the process of modeling real-life problems were organized into three main themes under the category of “mathematization”. First, the use of tables was prominent. Almost all participants (twelve students) stated that they preferred tables to organiza the data in an orderly manner, compare information, and manage the solution process more easily. This shows that students sought structural and visual support when dealing with complex data. For example, S1 said, *“I choose the table strategy because I can understand the data faster when I make it visual.”.* Second, eight students reported the use of figures and graphs to visualize the relationships between data and to concretize abstract structures. Number lines, figures, and graphs contributed to students’ understanding of the problem structure. S4 explained this as follows: *“I make tables or figures. These are very useful for me to understand the question.”*Finally, two students mentioned using calculators and computers as technological tools. These tools were preferred as they provided convenience in calculations and graphical representations. For example, S8 stated: “*First I make a table. Then I can also use a graph or computer.*” and S12 made the following statements: “*If there is a lot of data in the problem, I make a table. If there is a change in the data, I use a line graph. In this way, I see what increases or decreases*.”. In sum, students used visual tools such as tables, graphs and shapes more frequently than digital tools during the modeling process. These tools played an important role in understanding problems and developing solutions.

#### 3.2.4. Working mathematically.

The participant responses under the “Working mathematically” category were organized around two main themes: Model solution process and mathematical association strategies. In the model solution process, the participants adopted a systematic and step-by-step approach in problem solving. The majority of the participants (nine studens) preferred to do the operations in order, clearly specify the given and requested information, and use figures or tables when necessary. Proceeding step by step, checking for errors, and evaluating whether the result is logical were among the important components of this process. Some participants reporthe the use of technological tools such as calculators in the process steps. These statements revealed that the participants considered model solution not only as a calculation process, but also as a process of meaning and control. Some participants stated that they directly applied mathematical operations to situations they encountered in real life (e.g., supermarket discounts). This shows their understanding of mathematics’ functionality in daily life. In this theme, S12 stated, “*I draw a figure to explain the situation mathematically. Then I look at which numbers I use and perform the operation*.”. Similarly, S13 expressed “*When associating real-life situations with mathematics, I collect data from daily life and express them mathematically.”.* The participants statements regarding the strategies for associating real-life situations with mathematics showed students’ ability to analyze events and develop appropriate mathematical expressions. The participants (three students) first tried to understand what the event or problem was, and then thought about which mathematical knowledge they could use to solve it. In this process, methods such as observation, drawing shapes, converting to numerical expressions, and performing operations were prominent. For example, S1 explained, *“First, I try to understand what the event is about. Then, I think about which mathematical topic I can use to solve it.”.* In sum, students exhibited a structured, understanding and control-based approach in both model solving and mathematical association processes, and actively used strategies such as visualization, simplification and associating with real life.

#### 3.2.5. Interpretation and validation.

Participant statements regarding the interpretation and verification category were organized into four themes: interpreting the meaning of the model, evaluating its accuracy, identifying and correcting errors, and transferring models to different life situations. Eight participants stated that they focused on the daily equivalents of the numbers, operations, and expressions in the model to understand what the model represented in real life. While some students described the model as a small representation of a real-life event, others explained the model by justifying each step and explaining which information they used and how. For example, in this subject, S1 stated, *“I look at what each number and operation in the model represents in real life and try to understand it.”,* and S5 stated, *“I explain how I chose each step I took in the model according to the real-life situation. This way,* *everyone understands what the model is based on and what it represents.”.* Strategies such as reminding the problem, giving examples from daily life, and visualization were also frequently used. When evaluating how well the model explained the real-life problem, ten participants stated that they tested its accuracy by comparing their models with real-life data, questioned the model’s adequacy to solve the problem, and evaluated their understanding by explaining the model to their friends or teachers. This shows that both individual and social verification processes were used. For example, S2 said, “*I check the accuracy of my model by comparing it with a real-life problem. If everything is consistent, my model works well.*” and S10 said, “*I share my model with my friend to see if it works. If he/she can understand and apply the model, it is explanatory*.”. When an error was noticed in the model, most of the students (nine students) stated that they reviewed the solution steps from beginning to end, checked the operations or tried to fix the errors by correcting the incorrectly used formulas. For example, S4 explained, *“I check which step I made a mistake in and check the data. When I find errors, I correct that step using the correct data.”*. Similarly, S5 stated *“I check the calculations and formulas. If I made a wrong calculation somewhere, I correct that part.”*. Four students also stated that they preferred to correct the errors by redoing the solution. For example, S2 stated, *“If I notice an error, I solve it from scratch.”* and S13 reported *“When I notice an error in a model, I first review each step of the model. Then I correct the errors.”.* These statements showed that students’ self-control skills began to develop. Students’ ability to apply mathematical models to different life problems after the intervention was an important indicator of development. Twelve students stated that they could create models for many different daily life problems such as grocery shopping, water and electricity bills, traffic density, air temperatures, product efficiency, training times, vacation planning, saving money, and organizing a school trip. These examples revealed that students developed awareness in transferring their modeling skills to different contexts and relating them to daily life. For example, S3 stated, “*For example, I can use it for electricity and water bills at home. I can make suggestions for saving money.”* S5 stated*, “I can calculate the* *conditions under which the best crops grow by modeling the yield of different seed types, irrigation amounts, and climatic conditions.”,* S12 stated, *“I can model the changes in air temperatures in a city. Using daily temperature data, I can find the seasonal average temperature.”*. Similarly, S13 stated, *“I can develop a model to reduce traffic congestion in cities by using mathematical models to solve another real-life problem.”*. In sum, students’ explanations showed that they developed a holistic understanding of not only establishing the model, but also understanding, testing and reusing it.

## 4. Discussion

The aim of this study was to examine the impact of AI-pVR applications on students’ mathematical modeling competencies. VR technologies, which provide realistic renderings, have been shown to foster conceptual cognitive knowledge [56]. This study investigated the changes in mathematical modeling competencies of students who engaged with modeling problems through AI-pVR applications.

In this study, the AI-pVR application significantly enhanced the mathematical modeling competencies of gifted students. The VR environment provided students with a sense of immersion by allowing them to mentally engage with the problem and experience a simulation-based setting [57]. Furthermore, it helped create a meaningful learning environment through video visualizations that fostered understanding of mathematical concepts [58], which enabled students to perform deeper analyses and generate solutions to complex problems. In this sense, the application supported students in identifying real-life problems and developing solutions by applying their mathematical knowledge. In addition, students reflections further supported these findings. They indicated that the educational experience increased their attentiveness to their surroundings, enabling them to integrate mathematical thinking into various aspects of daily life. This shows that the connection they established between mathematics and real life became more multidimensional. These findings indicate that AI-pVR modeling processes not only fostered academic achievement but also contributed to the development of students’ environmental awareness and their ability to relate mathematical knowledge to real-world contexts.

The findings also showed that the AI-pVR application significantly enhanced students’ skills in problem understanding and simplification, which are critical components of mathematical modeling competencies. This finding is consistent with previous research showing that virtual reality-supported learning environments enhance students’ problem-solving flexibility and conceptual understanding through realistic and immersive elements [59]. In addition, our finding is in line with thosI n Khim Pham et al. [44] who reported that the use of virtual reality (VR) in teaching geometry improves mathematical modeling skills. The literature emphasizes that if the “problem understanding” phase—the initial step of the modeling cycle—is not properly structured, the effectiveness of the entire modeling process may be compromised [60]. In addition, simplifying complex real-world problems has been identified as a particularly challenging step for students [27,52]. Since the VR application creates a virtual learning environment that fosters students’ creativity by supporting flexible perception and enabling them to imagine things that do not exist in the real world [61,62], it helps students explore conceptual objects virtually and engage in virtual spaces that clarify abstract concepts [63]. These features allow students to interpret their environment more meaningfully, thereby raising their awareness of the problem, which in turn directly supports their capabilities to comprehend and simplify complex situations. In fact, the results of the present study demonstrated very clearly that the AI-pVR application fostered the development of these competencies. This finding is further supported by students’ reflections. Students stated that the intervention encouraged them to approach the problem-solving process more consciously. In particular, they reported improvements to their abilities to comprehend the problem, determine information of value when generating solutions to it, and think in a more systematic process. When students lacked complete information on a situation, they used their observations and logical assumptions to make simplifications, which is a more adaptable and strategic approach to problem-solving.

Furthermore, the AI-pVR application enhanced students’ mathematizing and working mathematically competencies—two essential components of mathematical modeling. This finding is consistent with Martin-Gutierrez et al. [64] who claimed that virtual learning environments supported students’ formalization processes by allowing them to make sense of the world while undertaking mathematical tasks. Similarly, Yang and Wang [65] reported that VR technologies allowed learning mathematical concepts to become easier as a result of the visualization of three-dimensional virtual objects. This study supports this point of view, with candidates reporting the video-based simulations they were exposed to during the training led to students’ abilities to conduct mathematical operations required for problem-solving. Campos et al. [66] and Soleimani [67] claimed that VR contributes to a deeper understanding of both basic concepts and problem-solving processes. In addition, students’ feedback further substantiated these results. They reported that the educational experience made them more inclined to use visual and digital tools functionally during the mathematizing process. This suggests that the integration of AI-pVR technologies not only facilitated students’ understanding of abstract mathematical structures but also fostered a more engaged and resourceful approach to transforming real-world problems into mathematical representations. Consequently, the use of visual models and simulations appears to strengthen students’ competence in applying appropriate mathematical procedures, thereby reinforcing their ability to work mathematically within complex problem contexts.

The study also found that the AI-pVR application significantly enhanced students’ modeling competencies in interpretation and verification. This result aligns with the findings of Steinberg [68], who suggested that VR applications improve students’ abilities to make predictions and interpret results, and Pelczer and Freiman [69], who indicated that real-life situations supported by virtual tools can help students generate and interpret results. Since this competency involves verifying and interpreting the applicability of mathematical results to real-life situations, it can be concluded that the VR application enhances this competency by allowing students to experience the problem as if it were real [70]. In virtual learning environments, concepts are constructed realistically and accurately; in addition, students can analyze problems more concretely by visualizing these concepts from a holistic perspective [71]. Given that the steps of interpreting solutions and verifying the model are considered critical in the modeling process [72], the development of these competencies is essential. Furthermore, the enhancement of students’ verification skills contributes to the development of their metacognitive processes during the modeling process [34]. The participant statements showed that the AI-pVR supported modeling process significantly improved not only the model building skills of the students but also their higher-level thinking and interpretation skills such as questioning the accuracy of the model, detecting and correcting errors, and transferring them to different life situations. In addition, the development of the students’ skills in associating mathematical models with daily life problems, applying them to new situations, and evaluating the validity of the model in multiple ways also revealed that the AI-pVR application made significant contributions to the integration of mathematical thinking with life in students.

## 5. Conclusions

Studies in mathematics education have demonstrated that mathematical modeling competencies are crucial factors in enhancing students’ academic success. The findings of this study revealed that the intervention had a significant large effect (η² = 0.37) on students’ mathematical modeling competencies. Among the components of modeling competence, understanding and simplifying the problem, mathematizing, working mathematically, interpreting, and verifying all exhibited significant large effects (η² = 0.33, 0.19, 0.32, 0.39, respectively), while identifying the problem showed a moderate effect (η² = 0.15). The variability observed in some measurements may be attributed to limitations in the number of practice trials and the small sample size. However, the educational process carried out within the scope of this study showed that students made significant progress in their mathematical modeling skills. Students stated that they meaningfully grasped the basic steps of the modeling process, such as analyzing real-life problems, relating these problems to mathematical structures, creating models, solving the model they created step by step, interpreting and verifying the results. In particular, students emphasized the use of tools such as tables, graphs and figures; planning the process steps, identifying and correcting errors, and relating the model to its real-life counterpart. In addition, students developed a significant awareness in sharing their models with their friends and teachers, receiving meaningful feedback, developing new models for different life situations, and establishing connections between real life and mathematics. These findings demonstrate that mathematical modeling-based instruction strengthens students’ mathematical thinking, problem solving, and relating them to daily life skills.

To draw more definitive conclusions, further research is required to examine the specific effects of presenting different task features with VR training on students’ cognitive and affective characteristics. Additionally, future studies should investigate the extent to which students can generalize the skills they acquire from VR to other learning scenarios outside the VR environment.

## 6. Limitation and future research

This study has some limitations. First, this research was limited to a school for gifted students located in a province in the western region of Turkey. Therefore, the generalizability of the findings is limited, and future research could include similar studies with participants from different regions and countries. Second, the data were collected from 60 6th grade students. Future research could compare results across students of different age groups and abilities. Third, the study was limited to an eight-week implementation period. Longer-term studies could be conducted to examine students’ mathematical modeling competencies. Fourth, this study used only local and touristic locations. Future studies could examine mathematical modeling competencies by designing modeling activities in different contexts. Fifth, the study used VR headsets and artificial intelligence tools. Different simulation software could be used in future studies. Finally, students were exposed to VR headsets for 20 minutes per session. Future studies could increase this duration to examine the effects on students’ motivation, creativity, and problem-solving approaches to mathematical modeling.
